# Case of Extensive Comminuted Fracture of the Cranium, with Recovery

**Published:** 1855-09

**Authors:** H. P. C. Wilson

**Affiliations:** Physician to the Baltimore City and County Alms House; Baltimore


*Case of Extensive Comminuted Fracture of the Cranium, with Recovery.* By H. P. C. Wilson, M. D., Physician to the Baltimore City and County Alms House.

We present the following case to the profession as one of peculiar interest. A boy, 8 years old, falls head foremost upon the pavement from the third story window of a house. The whole back part of the cranium is fractured and driven in upon the brain. Two inches square of bone are removed. The other fractured and depressed portions are accurately adapted, and the boy is well in four weeks from the receipt of injury. It is instructive in the lesson of the great amount of injury that may be received by the head and yet recovery follow; and as teaching a guarded prognosis in even the most desperate cases. It is no less instructive in the intimate relation between incipient ptyalism and returning consciousness. Whether it was post hoc and propter hoc I shall not attempt to say. The patient was treated actively with mercurials, and the result was most happy.

G. L., step-son of Mr. N., Saratoga street, Baltimore, aged eight years; health good, of robust constitution. Was called to see him May 31st, at 9 o'clock in the evening. Had just fallen from the third story window of a house upon the pavement.

I found him lying upon a sofa, icy cold, pulse rarely perceptible and intermittent, respiration slow, stertorous and irregular, pupils well dilated and unimpressible to light, perfectly insensible to all external impressions; has occasional emesis.—Examining the cranium, found fracture. No external wound except a small scratch. Back of the head completely flattened, the result of the blow.

I proceeded at once to cut down on what appeared to be the centre of fracture, which was about the point of union between the upper angle of the occipital and two parietal bones. I made a horizontal incision around the head, six inches in length, and another at right angles to the first, five inches; dissected back the flaps, and laid bare a comminuted fracture of the cranium, with the fractured portions driven in upon the brain. Dividing the periosteum, I removed two segments of bone, either of which was about one inch square. Four main fractures proceeded from this central point. One upwards and forwards across each parietal bone to about the parietal protuberance, (from either of which branched off several smaller fractures,) and two running downwards on either side of the middle line of the occipital bone, parallel to each other, and extending apparently to the foramen magnum. I did not venture to trace the whole extent of these fractures. The segment of bone dividing them was perfectly loose, so that seizing the upper extremity I was enabled to move it backwards and forwards with ease; but as it did not press upon the brain I desisted from any attempts to gratify curiosity, as to the extent of these fractures and the size of this segment. After removing the two pieces of bone mentioned above, I succeeded, without much difficulty, in inserting a loop under the remaining portions of the cranium pressing upon the brain, and with my finger as a fulcrum, dilated them and relieved their pressure. With the handle of my scalpel I removed all the accessible coagulated blood upon the dura mater, (this membrane being nowhere ruptured). I then drew the four flaps together at the point of intersection of the two incisions by two sutures, and applied no other dressings than a folded cloth dipped in cold water, and held in position by a bandage, thus allowing the incisions to pass freely, and give exit to any discharges that might be poured out from the seat of injury.

He was then removed to bed; hot water in bottles applied to his feet and body, and a little brandy administered from time to time. Reaction came on; pulse better; respiration easier. I left him for the night, with instructions that the dressings to his head should be kept wet with cold water: the brandy and bottles of hot water to be used or withheld, as the surface became cold or hot.

*June 1st.*—I am told this morning that our patient has spoken, and called persons by name, previous to my arrival. I find him however perfectly insensible, without any ability to be aroused. Pulse 120, small and quick; skin pleasantly warm; respiration easy; pupils dilated and unimpressible to light. Ordered hyd.

protochlo. grs. ij; potas. nitrat. grs. iv; pulv. ipecac. gr. $\frac{1}{4}$ ter die; as an antiphlogistic, and also as a sorbefacient to the bloody coagula upon the brain. To be fed with ice, which he took with great avidity. Head dressings to be undisturbed, and kept wet with cold water. Six o'clock P. M.—About as in the morning. Powders had given several alvine evacuations and vomited once; were discontinued, and friction with mercurial ointment substituted.

*June 2d.*—Still lying in a stupor, as if in a profound sleep. Pulse fuller and stronger, and 120. Ordered hyd. protochlo. grs. ij; potas. nitrat. grs. iv; acet. plumbi gr. $\frac{1}{2}$, ter die. Dressings removed for the first time. Nothing of note about the wound. Similar dressings reapplied, and same treatment continued.

*June 3d.*—Still in a stupor. Has shown no evidence of sensibility or consciousness of things around him since the morning after receiving the injury, and then it was only imperfect and temporary; but a flash of intelligence. To-day, however, I observe that his pupils, which have been uninterruptedly dilated, and without any impressibility to light, show evidences of contraction on passing a bright light before the eyes. Other symptoms unaltered; treatment unchanged; dressings undisturbed.

*June 4th.*—Pupils contracted and dilated freely under the influence of the presence or absence of light. Spoke on strongly arousing his attention, but at once relapsed into a comatose state, until another strong impression was made upon him. No evidences of inflammatory action about the wound. Water dressings re-applied and same treatment continued.

*June 5th.*—Found G. L. with his eyes open, taking notice of things around him. Answered when spoken to, and asked for what he wanted. Was lying very much as a child who is weary. Pulse 100, full but soft. Wound looks pale. No granulations thrown out, and no union taken place. Allowed crackers and tea and gave castor oil ℥ij. Previous treatment continued.

*June 6th.*—Still improving. Mind perfectly clear and evinces great intelligence. Playing with things around him. Is anxious for food. Complains, however, of sore mouth. Submaxillary glands a little tender, and increased secretion of saliva. Is slightly ptyalized. Has been taking calomel grs. ij. ter die, and using inunctions of mercurial ointment since the day after injury. Powders stopped, and the oil of yesterday, not acting, was repeated to-day. Is allowed some ice-cream, tea, toast and crackers. Solution of borate of soda, as a mouth wash. Wound looks pale and sickly. Stopped water dressings and applied a mutton-tallowed cloth and bandage. The cranium is seen bare through the gaping incisions.

*June 7th.*—Wound looks more healthy. Pulse good and 100. Shows much playfulness. Oil acted well. Is allowed soup, a soft-boiled egg, ice-cream, bread and tea. Other treatment unchanged.

*June 15th.*—Two weeks after the receipt of injury. A daily report has been omitted, because since June 7th the case has presented no symptoms worthy of note, and the treatment has been unchanged. Our patient has been going on steadily to improve. Is allowed a full nutritious diet. Wound dressed twice daily as heretofore. A good portion of it has healed by granulations, which are every where rapidly closing over the denuded bone. He is dressed and amusing himself with his playthings about the room.

*June 22d.*—Three weeks after injury. Is running about the house. Two arms of the crucial incision healed. Cranium visible but at one point, and here barely perceptible. Every where else covered by granulations. Has been on no treatment since 15th, except to dress head once daily as heretofore.

*June 29th.*—Four weeks after injury. Is no longer confined to the house. Meets me to-day on the pavement. Wound covered with cuticle, except a couple of small spots. Granulations being a little exuberant at these points, are touched with sulphat. cupri., and my patient discharged.

*July 26th.*—Saw G. L. on the street. Is perfectly well, with nothing to tell of his imminent peril and frightful injury but a firmly united cicatrix.

*Baltimore, August 8th,* 1855.

